# Local Inflammation, Dissemination and Coalescence of Lesions Are Key for the Progression toward Active Tuberculosis: The Bubble Model

**DOI:** 10.3389/fmicb.2016.00033

**Published:** 2016-02-02

**Authors:** Clara Prats, Cristina Vilaplana, Joaquim Valls, Elena Marzo, Pere-Joan Cardona, Daniel López

**Affiliations:** ^1^Departament de Física i Enginyeria Nuclear, Escola Superior d'Agricultura de Barcelona, Universitat Politècnica de Catalunya – BarcelonaTechCastelldefels, Spain; ^2^Unitat de Tuberculosi Experimental, Centro de Investigación Biomédica en Red de Enfermedades Respiratorias, Fundació Institut d'Investigació en Ciències de la Salut Germans Trias i Pujol, Universitat Autònoma de BarcelonaBadalona, Spain

**Keywords:** *Mycobacterium tuberculosis*, computational model, tuberculosis lesions in lungs, active tuberculosis, dynamic hypothesis

## Abstract

The evolution of a tuberculosis (TB) infection toward active disease is driven by a combination of factors mostly related to the host response. The equilibrium between control of the bacillary load and the pathology generated is crucial as regards preventing the growth and proliferation of TB lesions. In addition, some experimental evidence suggests an important role of both local endogenous reinfection and the coalescence of neighboring lesions. Herein we propose a mathematical model that captures the essence of these factors by defining three hypotheses: (i) lesions grow logistically due to the inflammatory reaction; (ii) new lesions can appear as a result of extracellular bacilli or infected macrophages that escape from older lesions; and (iii) lesions can merge when they are close enough. This model was implemented in Matlab to simulate the dynamics of several lesions in a 3D space. It was also fitted to available microscopy data from infected C3HeB/FeJ mice, an animal model of active TB that reacts against *Mycobacterium tuberculosis* with an exaggerated inflammatory response. The results of the simulations show the dynamics observed experimentally, namely an initial increase in the number of lesions followed by fluctuations, and an exponential increase in the mean area of the lesions. In addition, further analysis of experimental and simulation results show a strong coincidence of the area distributions of lesions at day 21, thereby highlighting the consistency of the model. Three simulation series removing each one of the hypothesis corroborate their essential role in the dynamics observed. These results demonstrate that three local factors, namely an exaggerated inflammatory response, an endogenous reinfection, and a coalescence of lesions, are needed in order to progress toward active TB. The failure of one of these factors stops induction of the disease. This mathematical model may be used as a basis for developing strategies to stop the progression of infection toward disease in human lungs.

## Introduction

In general, the first step of the natural history of tuberculosis (TB) is characterized by the infection of alveolar macrophages (AMs) with *Mycobacterium tuberculosis* (Mtb). The bacilli are able to grow intracellularly until they destroy the AM and are then able to infect neighboring cells from close alveolar spaces. As Mtb is a slow grower, with a doubling time of around 24 h, this process lasts for around 1 week (Cardona, 2010). Destruction of the AM can be controlled by the AM itself through a process of apoptosis, or blocked by the bacilli, causing necrosis of the AM. This is a very important process as it determines both the intracellular bacillary concentration tolerated by the AM and the inflammatory response triggered (Lee et al., 2006). This process is also known to be influenced by the initial bacillary load phagocytosed by the AM, with a higher load increasing the probability that necrosis is induced (Lee et al., 2006). Data from different experimental models in laboratory animals indicate that the immune response appears 2–3 weeks after low-dose aerosol challenge (Vilaplana et al., 2014). This is a Th1 cellular immune response, thus requiring a proliferation of specific lymphocytes in hilar lymph-nodes (North and Jung, 2004) that will be attracted toward the infected site if a sufficiently high inflammatory response is triggered to do so (Bru and Cardona, 2010; Cardona and Ivanyi, 2011; Vilaplana et al., 2014).

In the majority of cases this response is sufficient to control the infection, which results in lesions with a diameter of < 1 mm that finally encapsulate and are reabsorbed or calcify, thus killing the bacilli in the necrotic tissue (Gil et al., 2010). It has been demonstrated that drainage of the bacilli is possible until this encapsulation process takes place, thus allowing an endogenous reinfection of the AM that is responsible for maintaining the latent tuberculosis infection (LTBI), although the majority of drainage occurs via the gastrointestinal duct, thus finally resulting in complete elimination of the bacilli (Cardona, 2010). Equally, the window of opportunity between AM infection and the attraction of specific lymphocytes to the infection site allows exogenous reinfection even once the immune response is set, thus having a marked influence in countries with a high TB incidence (Cardona and Vilaplana, 2014). All in all, this process does not hamper the health status of LTBI subjects. The problem is that in a small percentage of cases (around 10%), there is no infection control and the lesions reach a size of at least 10 mm, thus causing the TB to become active. There is currently no clear explanation of the mechanisms responsible for this.

Recent data obtained by different groups and using different experimental approaches have demonstrated a key role for neutrophils in the induction of TB (Berry et al., 2010; Eum, 2010; Green et al., 2010). The murine model using C3HeB/FeJ has provided a great deal of information in this respect as it is possible to reproduce human-like lesions, inducing liquefaction thanks to its ability to accumulate neutrophils (Yan et al., 2006; Driver et al., 2012; Harper et al., 2012; Irwin et al., 2014; Marzo et al., 2014). We have been able to demonstrate that this process requires the accumulation of different lesions that, once coalesced, can induce TB lesions quickly enough to avoid the encapsulation and isolation of small lesions (Marzo et al., 2014; Vilaplana and Cardona, 2014). A detailed review of human lesions in the pre-antibiotic era has also confirmed this view (Cardona, 2015).

Despite all the experimental observations discussed above, the natural history of tuberculosis is not yet fully understood. In particular, the factors that cause a latent infection to evolve toward active disease still form part of a conceptual hypothesis that is far from being demonstrated. Consequently, a great deal of research effort has been expended in this direction and the use of models that could help to unravel these dynamics is, therefore, essential.

A model is a simplified representation or description of a system. The degree of complexity, i.e., factors and processes to be taken into account, and the spatio-temporal scale depend on the question that the model aims to address. The kind of modeling form and strategy must also be chosen accordingly. In fact, mathematical models can be classified according to different criteria (e.g., empirical or mechanistic, spatially explicit or implicit, and continuous or discrete, amongst others; Haefner, 2005). The strategy used to build them may be either top-down or bottom-up. A top-down strategy would cover, for instance, the description of a certain system's dynamics by means of global differential equations. This strategy is useful and powerful when the heterogeneities of the system are not relevant to the problem addressed, and if the variables addressed fit the continuum hypothesis. The bottom-up strategy requires the parts of the system, and the interactions among them, to be modeled in order to reproduce the whole-system dynamics. This is an interesting approach whenever emergent behaviors are expected to arise. Individual-based and Agent-based models (AbM) are examples of bottom-up computational approaches. Grimm (1999) defines AbM as “simulation models that treat individuals as unique and discrete entities which have at least one property in addition to age that changes during the life cycle.” They have been widely used in ecology and in microbiology (Kreft et al., 2013).

In the past decade, the use of mathematical modeling in TB research has increased a great deal (Kirschner et al., 2010; Zwerling et al., 2015). These models aim to describe the dynamics of tuberculosis infection on different spatio-temporal scales, from the molecular-intracellular level up to the epidemiological scale, covering the granuloma, the tissue, and the different organs involved, among others (Guzzetta et al., 2011; Kasaie et al., 2013; Gong et al., 2015; Linderman et al., 2015). Depending on the problem addressed, bottom-up, top-down, or hybrid approaches have been used as modeling strategies (Bru and Cardona, 2010; Vilaplana et al., 2014; Cilfone et al., 2015). Mathematical models have been shown to be an essential tool to interpret experimental results and disentangle tuberculosis natural history (Kirschner and Linderman, 2009).

This paper is focused on a spatial scale slightly superior to granuloma. In fact, we propose a study of the spatial relationship between close TB lesions. This is an important issue in the effort to improve understanding of the connection between properties of neighboring granulomas and their extension through the lung. To do this, we have built an AbM aimed at understanding experimental information from an animal model. In particular, the purpose of this paper is to evaluate the role of local inflammation, dissemination and coalescence of lesions in the progression to active disease in a mice model by means of an AbM.

## Materials and methods

### Summary of previous experimental results

**Figure 1:**
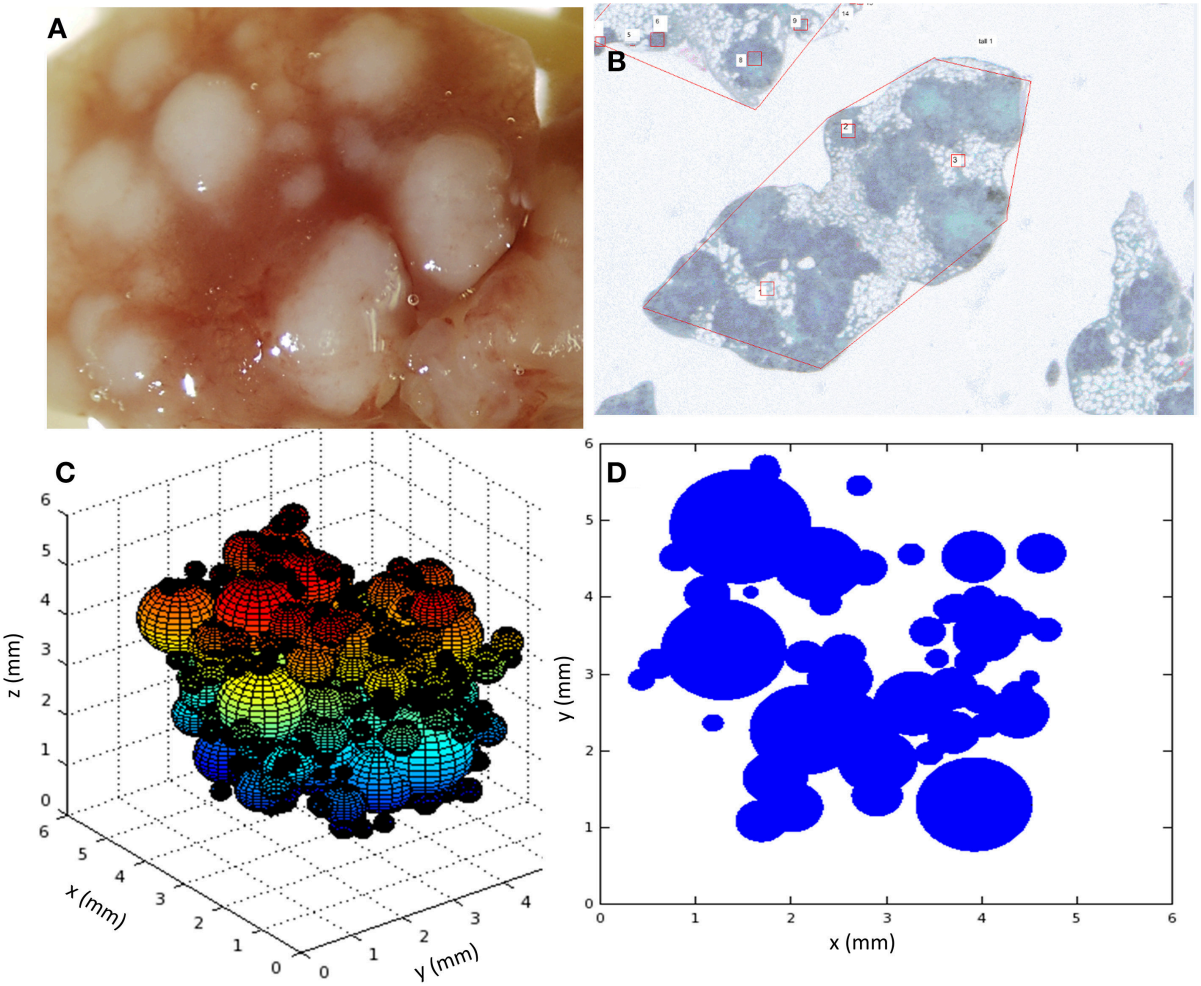
**(A)** Macroscopic image of the lung at day 30; **(B)** Microscopic image of *paraffined* slides of infected mice lungs; **(C)** snapshot of a 3D view of the simulation system, each sphere representing a lesion; **(D)** snapshot of a 2D slide of the simulation system, each circle representing a slide of a lesion. Simulations used parameters of panel B (**Table 1**).

Marzo et al. (2014) reported several series of experiments in which the evolution of tuberculosis lesions in C3HeB/FeJ female mice was studied. Six-eight-week-old specific-pathogen-free mice were infected with 2 × 10^4^ Colony Forming Units of *M. tuberculosis* H37Rv Pasteur strain via the caudal vein. They were then euthanized at various time points in order to analyze different parameters related with TB infection dynamics (number and/or size of tuberculosis lesions, and total affected area, among others). Six animals were included per time point. A macroscopic image of lesions in a mouse lung obtained in these experiments is shown in Figure 1A.

Some of these experimental results were used to establish the basis of the mathematical model we present here and to calibrate it. In particular, we focused on those experiments related to the evolution of the number of tuberculosis lesions and affected area in infected mice lungs. These measurements were obtained by microscopy after fixing lungs in 10% buffered formalin, embedding lung lobes in paraffin, cutting them into 5-μm slides, and staining them with Masson trichromic (MTC). An example of a microscopy image taken from an MTC-stained recut is shown in Figure 1B. One of the reported series that will subsequently be denoted as series A aimed to track the temporal evolution of the number of tuberculosis lesions and total affected area. Another of the experimental series that will subsequently be denoted as series B was focused on the area distribution of lesions on a specific day.

In series A, measurements were carried out on days 21, 23, 26, 28, 30, 31, 32, and 33 post-infection in order to study the dynamics of the experimental model. The number of animals for each sampling day was variable (from 1 to 6). The observations that are of interest for the present study were:

1. The dynamics of lesions depended on the mice species and on the treatment applied. In this study, we will focus on the behavior of the infection in female C3HeB/FeJ mice (6–8 weeks old) without any specific drug treatment.
2. C3HeB/FeJ is an animal model of active TB that reacts against *Mycobacterium tuberculosis* with an exaggerated inflammatory response. Experimental measurements showed that the affected area increased exponentially.
3. The number of observed lesions initially increased, thereby suggesting that a few bacilli or infected macrophages can escape from mature lesions and generate new infection foci.
4. In addition, after a few days the number of lesions started fluctuating. At this point, microscopy observations allowed the identification of big lesions comprising several sub-lesions, thus suggesting a coalescence phenomenon between lesions that had come into contact.

These experimental results are presented graphically in the first figure of the cited article (Marzo et al., 2014).

In series B, measurements were carried out on days 21 and 28 to specifically determine the individual areas of all detected lesions and thus obtain the lesion area distributions. In this case, the mean lesion area was 0.266 mm^2^ at day 21 and 3.36 mm^2^ at day 28. The area distribution at day 21 followed an exponentially decreasing shape.

### The mathematical model: Bubble model

This model was built with the aim of understanding the experimental results mentioned above and, therefore, to suggest an objective explanation of the dynamics observed. The main features to be taken into account were that (i) affected area increases almost exponentially; (ii) the number of lesions initially increases and then fluctuates; and (iii) the lesion area distribution follows a negative exponential at day 21, with the exponent of the fitting being the inverse of the mean area. The first and second observations were considered in the model design, and the third one was expected to emerge as a result of model simulations.

Given this context, we chose the bottom-up AbM strategy as the most appropriate, the lesion being the agent to be modeled. Therefore, the lesion was taken as a black box whose individual behavior is the result of processes on other scales that are not specifically modeled. According to the experimental information, the temporal scale was fixed at a few weeks.

The hypotheses used to construct the model were as follows: (1) lesions grow logistically due to the inflammatory reaction, the maximum being given by physical space limitations; (2) new lesions can appear as a result of the escape of extracellular bacilli or infected macrophages from older lesions, according to the endogenous reinfection theory; and (3) lesions can merge when they are close enough, showing a coalescence behavior. The third hypothesis is why we call this model the “Bubble model,” as preliminary versions were inspired on the coalescence between soap bubbles which, like the lesions observed, are spherical and have an internal overpressure that is inversely proportional to their radius. This overpressure inside a bubble (Δ*P*) is governed by the Young-Laplace equation, which states, that Δ*P* = 2·σ·*r*^−1^, σ being the surface tension and *r* being the radius of the spherical bubble. This relationship between radius and pressure causes two bubbles to fuse when they come into contact as the air in the smallest bubble/lesion (with a higher overpressure) is pushed into the bigger one (with a smaller pressure inside; Escolar and Escolar, 2004).

As mentioned above, the lesions were taken as the main entities of the model and treated individually, as in an agent-based model (Ferrer et al., 2008). This kind of model allows the study of macroscopic emergent behaviors on the basis of agents' dynamics and interactions. The variables assigned to each lesion included their position in a 3D space (*x*_*i*_, *y*_*i*_, and *z*_*i*_, in mm), their size (radius *r*_*i*_, in mm), their area in the intersection with a certain plane *j* (*A*_*i, j*_, in mm^2^), in an attempt to mimic their area in experimental cuts, and their age, expressed as the time since apparition (*T*_*i*_, in days). The subindex *i* refers to a certain lesion of the system. According to experimental observations (Marzo et al., 2014), all lesions were considered as spheres.

**Figure 2:**
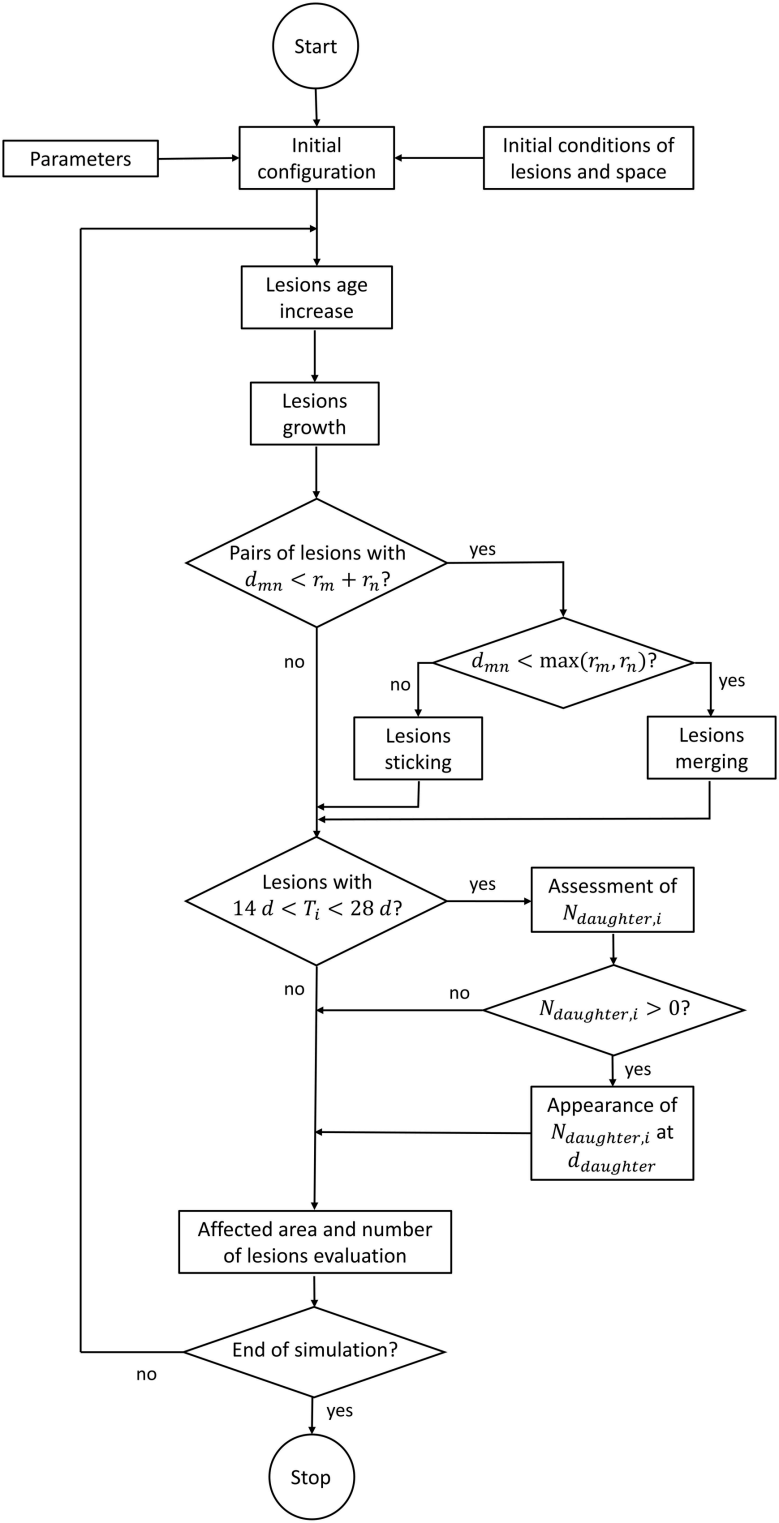
**Flow chart of the computer code**.

The processes that drive the dynamics of these individual lesions were modeled as follows (Figure 2). Most of parameters of the mathematical functions used for describing such processes were affected by a certain noise, where indicated.

#### Lesion position and age

Lesions were considered to be fixed in space, with their center at the spatial point that is initially assigned to them. Their age was considered to increase linearly with time.

#### Lesion growth

In general, an increase in lesion radius would initially be driven by an increase in its bacterial load and subsequently modulated according to the inflammatory response (which would accelerate such an increase) and the immune response (which could accelerate or decelerate this increase, and even produce a decrease in volume). In the case of C3HeB/FeJ mice, the inflammatory reaction is exaggerated. Consequently, the effective growth of lesions was modeled through a logistic growth in the surface of the lesions *S* (Haefner, 2005), as this growth is mainly result of an accumulation of cells in it (Equation 1):

(1) $$\begin{array}{r} \frac{dS}{dt}=\alpha \;\cdot \;S\;\cdot \left(\;1-\;\frac{S}{S_{max}}\right) \end{array}$$

*S*_*max*_ and α would be the maximum surface and growth rate, respectively. If we want to deal with the radius as the main variable, we can re-write Equation (1) to give Equation (2):

(2) $$\begin{array}{r} \frac{d\left(4\pi r^{2}\right)}{dt}=\alpha \;\cdot \;\left(4\pi r^{2}\right)\cdot \left(1-\left(\frac{4\pi r^{2}}{4\pi {R_{max}}^{2}}\right)\right) \end{array}$$

Consequently, the equivalent growth curve for a particular lesion's radius *r*_*i*_ was modeled as shown in Equation (3).

(3) $$\begin{array}{r} \frac{dr_{i}\left(t\right)}{dt}=\;\nu_{\;i}\;\cdot \;r_{\;i}\left(t\right)\cdot \left(\;1-\;{\left(\frac{r_{i}\left(t\right)}{R_{j,i}}\right)}^{2}\right) \end{array}$$

In simulations, the constant *v*_*i*_ (in day^−1^) was randomly assigned to each lesion using a normal distribution around a mean value, $\bar{v}$. This mean value was fixed after a parameterization process. The existence of a maximum radius (*R*_*j, i*_) is due to spatial (physical) and biological limitations, and it was allowed to acquire two different values below (*j* = 1) and above (*j* = 2) a certain threshold (*r*_*thres, i*_ = *R*_1, *i*_), in order to allow the growth of large, coalesced lesions (i.e., an individual lesion could grow to a certain value, *R*_1, *i*_, never reaching this maximum asymptotic value, but a large lesion arising from the coalescence of two or more lesions could grow to a larger radius, *R*_2, *i*_).

#### Lesion coalescence

The joining of two neighboring lesions (e.g., *L*_*m*_ and *L*_*n*_) was modeled as a two-step process. When the distance between their centers, *d*_*mn*_, is lower than the sum of their radii, *r*_*m*_+*r*_*n*_, they keep growing individually but are considered as one unit (with two sub-lesions) in the total count. Once this distance is less than the largest radius, either *r*_*m*_ or *r*_*n*_, an effective merging between them is considered. In this case, the radius of the joined lesion is equal to $\sqrt[{3}]{{r_{m}}^{3}+{r_{n}}^{3}}$, (i.e., it is equal to the radius of a sphere whose volume is the sum of the volumes of the two constituent spheres) and its center is located at the same point as the center of the previous biggest lesion.

#### Daughter lesions

In the model, a lesion with an age of between 14 and 28 days was supposed to be able to generate other lesions. We assumed a model based on a certain probability of generating a new lesion as a function of *mother*'s age and radius. This probability of generating new visible lesions was considered to decrease linearly with the *mother*'s age (*T*_*i*_(*t*)) and increase linearly with radius (*r*_*i*_(*t*)). Thus, the number of daughter lesions emerging from a particular lesion at a certain time, *N*_*daughter, i*_(*t*), is determined as follows (Eq 4):

(4) $$\begin{array}{rcl} N_{daughter,i}\left(t\right) & = & \rho_{\;i}\;\cdot \;\frac{r_{i}\left(t\right)}{r_{min}}\;\cdot \left(\;2\;-\;\frac{T_{i}\left(t\right)}{14\text{days}}\right);\\ \forall {\;14days\;<\;T}_{i}\left(t\right)<\;28\;days \end{array}$$

The resulting number is truncated in order to provide an integer amount of daughters that can be *N*_*daughter, i*_(*t*)≥0. The parameters ρ_*i*_ (adimensional) and *r*_*min*_ (in mm) are an individual probability resulting from a mean probability ($\bar{\rho }$) and a minimum radius, both of which are fixed in the parameterization process. The distance from the mother lesion at which daughter lesions appear (*d*_*daughter*_, in mm) was considered to decrease exponentially (Equation 5).

(5) $$\begin{array}{r} d_{daughter}=\delta_{0}\cdot \Gamma \left(10,0.1\right) \end{array}$$

Γ(10, 0.1) is the mathematical gamma distribution with the appropriate parameters for describing a negative exponential. The constant δ_0_ (in mm) was also determined during the parameterization process.

### Model implementation and scheduling

The mathematical model was implemented in Matlab® in order to run it, evaluate its behavior and determine the role and value of the most important parameters. The time step was fixed at half a day, and the 3D space was fixed as a cube with an edge of 8 mm. These spatial dimensions were chosen according to the real size of analyzed lungs. Nevertheless, since we lacked 3D spatial structure data about these lungs, we decided not to provide spatial-specific structure to the model and we used the cube for simplicity.

The need to compare the results obtained with microscopy measurements required the implementation of a specific protocol to analyze simulation results. Although the model was run in a 3D space where lesions were considered as spheres, the results were analyzed on a random 2D slide of this 3D system, as shown in Figure 1. This emulates the experimental procedure consisting of cutting the lungs into slides and analyzing these slides with the microscope. In fact, another key point to take into consideration when comparing simulations with experiments arises from this lack of spatial data about each experimental slide, i.e., we do not know where this slide was located in the original lung. This is why the simulation slide for analysis was randomly chosen. The consequence is that it was not possible to compare the quantitative behavior of experimental and simulation extensive variables, which depend on the global size of the system. Instead, intensive variables (i.e., those that do not depend on the slide size) had to be used for this purpose.

It is important to note that most of the parameters in the model were affected by Gaussian noise, as mentioned above, such that they could vary slightly from lesion to lesion, from time to time, and/or from mouse to mouse. This stochasticity allows the simulator to provide the variety of behaviors observed in all experimental measurements with a single model. In fact, each individual simulation was considered as emulating one mouse. Simulation series were therefore scheduled according to the number of experimental measurements in each case.

Another important point related with model implementation arises from the fact that lesions are not observable by microscopy until they have a diameter of 0.15 mm, which occurs at the age of 14 days, approximately. Thus, the simulations started with observable lesions at day 14. This is equivalent to considering the lesions from the moment at which they can be measured experimentally.

The simulation schedule involved three main steps or series of simulations: (i) an initial set of simulations with preliminary parameters in order to test the qualitative behavior of the model; (ii) a series of simulations specially designed for carrying out a sensitivity analysis and parameter estimation; and (iii) a series of simulations to validate and test the implications of the model.

#### Initial conditions

The initial conditions for simulations were fixed as follows. The initial number of lesions was set at 50 or 100, depending on the simulation series. All of them were randomly distributed in the considered space, and their initial age and size were assigned as 14 days and 0.15 mm, respectively, with Gaussian noise.

### Sensitivity analysis and parameter estimation

The sensitivity analysis and parameter estimation followed an iterative process that entailed successive simulations in order to (i) delimit the ranges where parameters and outcome results made biological sense, and (ii) identify those parameters that were more sensitive. Once these ranges were fixed, we designed a final series of simulations to perform a grid search within these intervals and find the best values for the most sensitive parameters.

As a result of the initial scanning, we found that the most relevant parameters were the mean growth rate of lesions, $\bar{v}$, the individual mean probability for determining the number of daughters, $\bar{\rho }$, and the mean distance at which daughter lesions appear, δ_0_. Then, in order to estimate the best values, we chose the following objective function (Equation 6) as indicator on the basis of the experimental information available.

(6) $$\begin{array}{r} F_{obj}=\sqrt{\sum {\left({\bar{A}}_{\;exp,t}-{\bar{A}}_{sim,t}\right)}^{2}} \end{array}$$

${\bar{A}}_{exp,t}$ and ${\bar{A}}_{sim,t}$ correspond to the mean area of lesions in a slide measured in experiments (*exp*) and simulations (*sim*) at different time points *t*. As we worked with two experimental series, we estimated two parameter sets in order to minimize the corresponding objective functions. The values obtained are shown in Table 1. The differences between the values obtained for series A and B are in the range of experimental variability, which was relevant.

**Table 1:** **Parameters of the model after the parameter estimation process**.

**parameter**	**Description**	**Value (A)**	**Value (B)**	**Units**
$\bar{\nu }$	Mean value for growth parameter (Equation 3)	0.17	0.14	day^−1^
${\bar{R}}_{1}$	Mean maximum radius before merging (Equation 3)	1.0	1.0	mm
${\bar{R}}_{2}$	Mean maximum radius after merging (Equation 3)	20	10	mm
$\bar{\rho }$	Mean value for daughter lesion probability (Equation 4)	0.8	0.8	–
${\bar{r}}_{min}$	Mean minimum radius of lesions (Equation 4)	0.15	0.15	mm
δ_0_	Constant for determining the distance at which a daughter lesion appears (Equation 5)	1.5	1.0	mm

*Columns A and B are parameters obtained from fittings to experimental series A and B, respectively*.

## Results

### The Bubble model explains the general dynamics of lesions

Figure 1 shows two snapshots of the simulations (Figures 1C,D) together with two images taken from the experimental results (Figures 1A,B). As mentioned above, a key point of the comparison between experiments and simulations is that the microscopic analysis was performed in randomly selected *paraffined slides* of the infected lungs. In fact, as can be seen in Figure 1, the number of lesions in the lung as a whole may be between one and two orders of magnitude higher than the number lesions observed in a single slide. In addition, there is a lack of information about the position of the experimentally analyzed slides in the original lung. Thus, it is not possible to extrapolate the quantitative behavior of extensive variables like the total affected area, since the geometrical constraints of experimental measurements were not homogeneous. In order to calibrate and validate the model, we decided to use intensive variables like the mean area of lesions, which are independent of the geometry of the slide considered.

**Figure 3:**
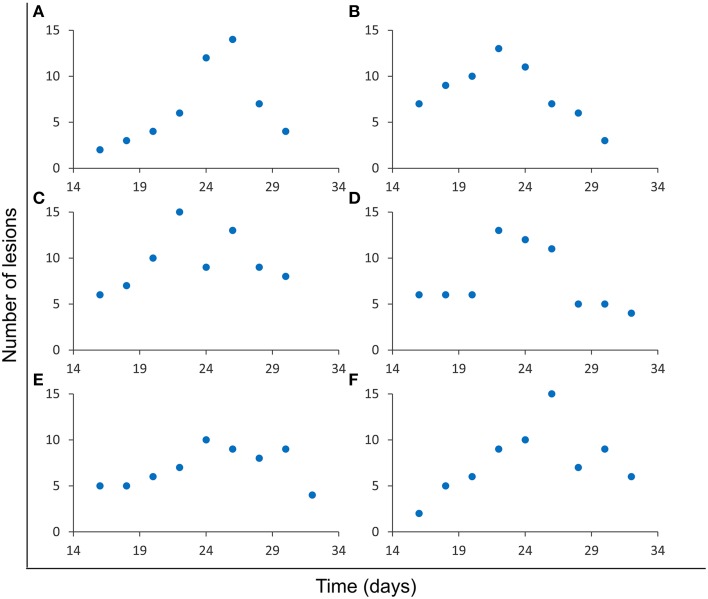
**Evolution of the number of lesions in a single slide**. Results from six independent simulations **(A–F)** obtained using the parameters for series **A (**Table 1**)**. Different fluctuation patterns can be observed for this variable.

All simulations reported fluctuations in the number of lesions in an intersection plane *j*. The number, intensity, and slope of these fluctuations depended on the simulation and the slide concerned, as can be seen in Figure 3. The variety of patterns observed include single (Figures 3A,B,D) and multiple (Figures 3C,E,F) fluctuations, slight (Figure 3E) and deep (Figure 3A) fluctuations, or smooth (Figure 3B) and abrupt (Figure 3D) fluctuations, amongst others.

**Figure 4:**
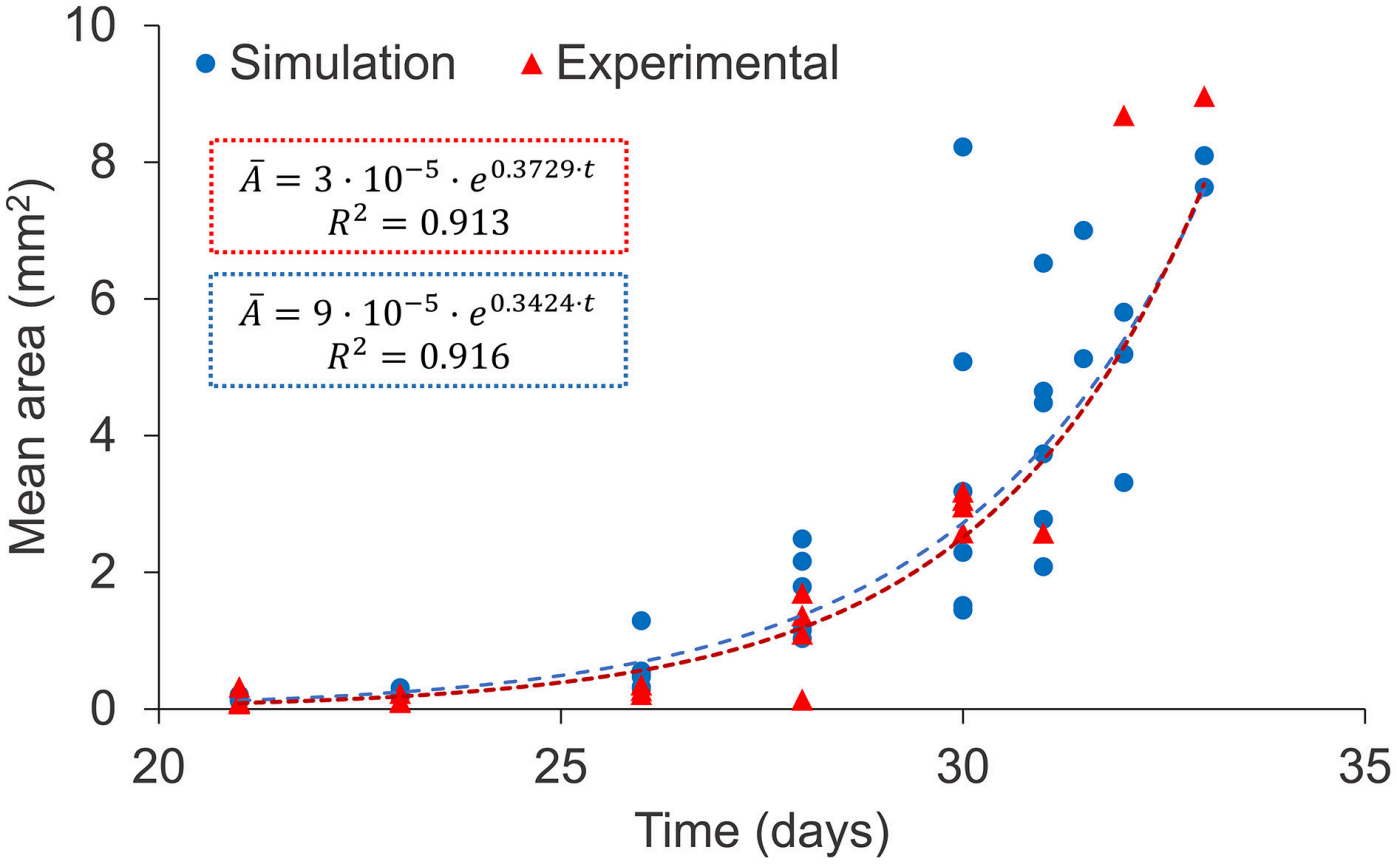
**Evolution of the mean area of lesions: experimental data (red triangles) and simulation results (blue circles), both corresponding to series A (Table 1)**. Exponential fittings to simulation results and experimental data are shown (dotted lines). Two outlying experimental data-points were not taken into account for the fitting.

As mentioned above, the evolution of the total affected area cannot be compared numerically since spatial dimensions are not homogeneous between lung samples. As such, the variables that can be quantitatively compared are those which are intensive, as is the case of the mean area of lesions. Both the experiments and the simulations showed an exponential increase in this variable, as can be seen in Figure 4. Exponential fittings to both data series provided similar values for slopes and independent terms, as well as good correlation coefficients (*R*^2^ > 0.9).

### The negative exponential distribution of areas meets the maximum entropy principle

**Figure 5:**
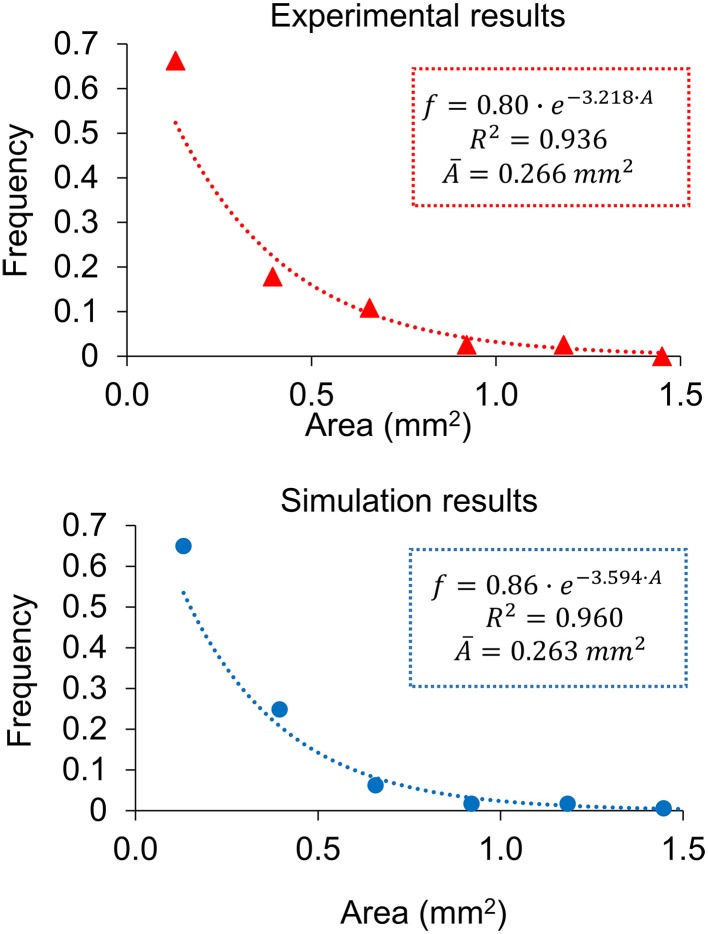
**Distribution of individual lesion areas at day 21 for the experimental data (red triangles, up) and the simulation results (blue circles, down), with the exponential fittings**. Both correspond to series B.

A proof of consistency of a model may arise from the fact that results that have not been looked for are in accordance with experimental observations. In this case, an analysis of the experimental results for series B (Marzo et al., 2014) suggests that the areas of individual lesions at day 21, when the infection is still far from fully occupying the lungs, are exponentially distributed. At this point, the system has been allowed to evolve without specific physical constraints. An analysis of the simulation results at day 21 also shows an exponential distribution of the area distribution. This was not specifically imposed on the model and was not taken into account for calibration, thus meaning that it is a proof of consistency. Figure 5 shows both distributions with exponential fittings and regression coefficients, which are *R*^2^ > 0.9 in both cases. The constants fitted to the simulation results have the same order of magnitude as those fitted to experimental results (11% deviation in the exponent and 7% in the intersection).

Now let us consider the distribution of a specific variable among the components of a system (e.g., in this case the variable concerned is the mean area, and the distribution gathers the frequency of lesions in each area interval, as shown in Figure 5). The diversity of this system according to this variable can be assessed using Shannon's diversity index, $H=-k\underset{\;i}{\sum }p_{\;i}\;\log\;p_{\;i}$ (*k* = 1∕log2), with *p*_*i*_ being the frequency of the system's components in each bin of the distribution considered according to the maximum entropy principle, the maximization of this index with the appropriate constraints provides the distribution that maximizes the diversity of the system. The exponential distribution is characteristic of a system that maximizes entropy with the only constraint of limited energetic resources (Ferrer et al., 2011).

In the case of lesion growth in the infected mice, the results show that while inflammation can evolve without more constraints, the system tends to a maximum diversity (exponential) distribution. In addition to the correlation coefficients, an indicator for a distribution to be exponential is that the exponent of the curve is equal to the inverse of the distribution's mean value. A detailed analysis of the experimental distribution provides an exponent of 3.218 mm^−2^, with its inverse (0.31 mm^2^) being close to the mean area of 0.27 mm^2^ (13% deviation). Similarly, the simulation results provide an exponent of 3.594 mm^−2^, the inverse of which (0.28 mm^2^) is also close to the mean value of the area (0.26 mm^2^, 5% deviation). Once the infection gets totally out of control and the affected area achieves a high value, the physical limitations of the lungs add new constraints and the area distribution is no longer exponential, as occurs at day 28.

### Local inflammation, dissemination and coalescence of lesions are key for the progression toward active tuberculosis

**Figure 6:**
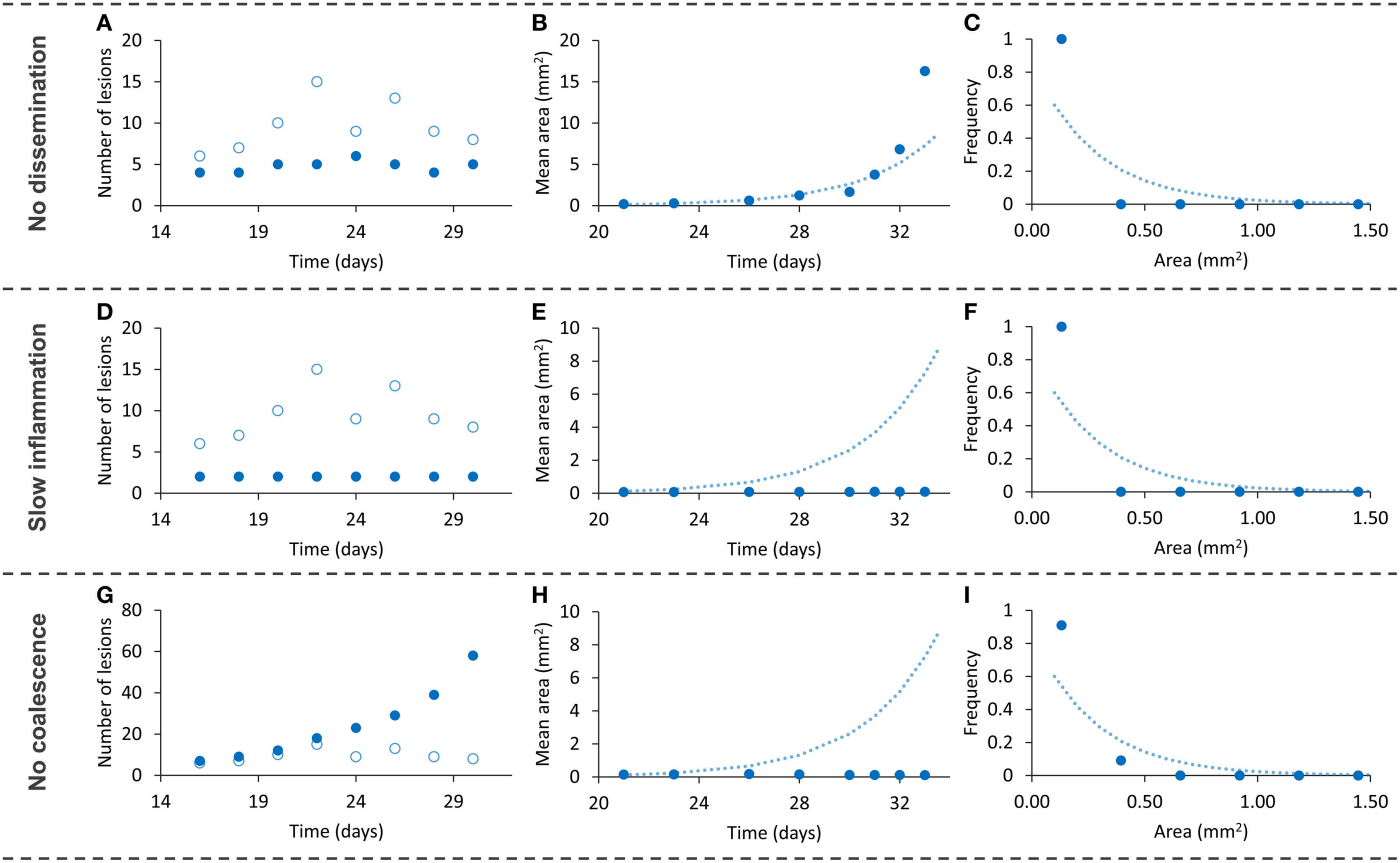
**Dynamics of the three variables studied (number of lesions—left, mean area evolution—center, area distribution at day 21—right) when removing one of the hypotheses from the model [no dissemination—top (A–C), slow inflammatory response—middle (D–F), no coalescence—bottom (G–I)]**. Light empty symbols and dotted lines correspond to the results obtained with the complete model, using parameters of panel A (Table 1). Figures 6E,H are enlarged in Figure 7.

Finally, we designed an additional series of *in silico* experiments to assess the importance of the model's hypotheses. To this end, we individually removed each hypothesis from the model and ran a simulation. We considered no endogenous reinfection in the first run (no daughters), a 10-fold decrease in the intensity of the inflammatory response in the second one (slow inflammation) and an absence of coalescence in the third one (no coalescence). We studied the effects on the already reported variables (number of lesions in a slide, evolution of the mean area and distribution of individual lesions' areas at day 21). The obtained outcomes are shown in Figure 6.

The absence of endogenous reinfection provides the expected stability in terms of number of lesions, with the small increase in this value being due to the growth of a lesion from a neighboring intersection plane and slight decreases being related to coalescence between lesions. In this case, the mean area increases to higher values with respect to the previous simulations, as can be expected if the number of lesions remains approximately constant and the lesion area keeps increasing. The area distribution at day 21 suggests that endogenous reinfection is a necessary mechanism for lesions to grow via coalescence: if no new lesions appear, fewer fusions between neighboring lesions take place and the dominant mechanism for them to grow is the inflammatory response.

**Figure 7:**
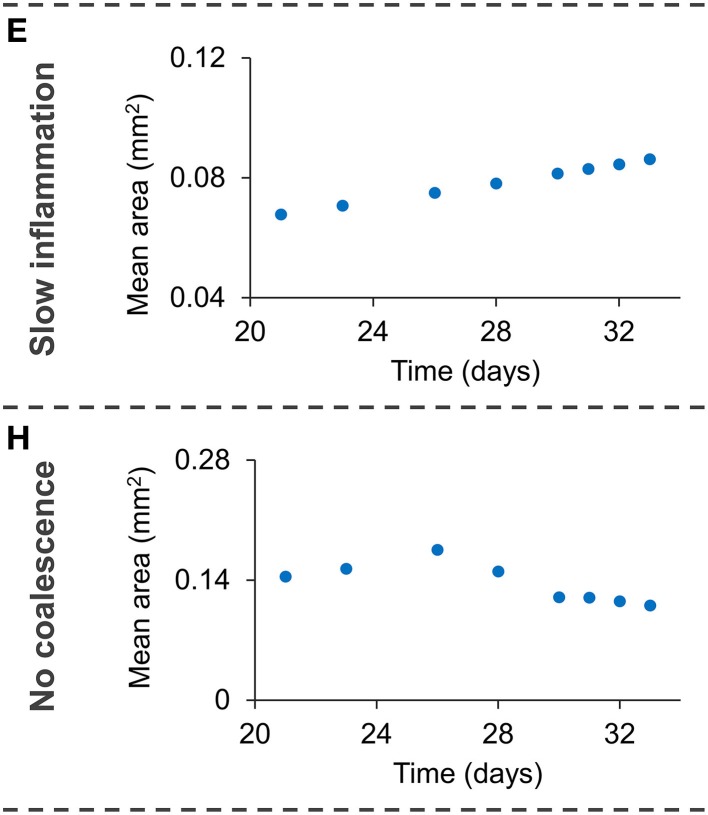
**Enlargement of Figures 6E,H, showing the small increase in mean are with a small inflammatory response (E) and with no coalescence (H)**.

A 10-fold decrease in the intensity of the inflammatory response markedly reduces the dynamics of the system. In fact, and as can be observed in Figure 7, the increase in mean area exists but is very small. As a consequence, the lesions are unable to fully develop and generate new lesions. The third run demonstrates that, in addition to the inflammatory response, coalescence is an essential mechanism for lesions to grow. Even though the number of lesions increases constantly, their mean size remains smaller than in the bubble model simulations (Figure 7).

## Discussion

We have built a mathematical model that takes into account three processes that may be considered key factors for the progression of a TB infection toward active disease, namely high inflammatory response, endogenous reinfection, and coalescence between lesions. This model was implemented in a computer code in order to validate and quantify the role of these processes. A confirmation of the bubble model's plausibility is that it considers the individual dynamics and interactions of lesions, and the macroscopic behavior emerges. Thus, the positive results we obtained demonstrate the essential role of local inflammation, endogenous reinfection, and coalescence, as shown in Figure 6, and opens up new perspectives in the natural history of tuberculosis. In fact, using the bubble model we have transformed theories such as the dynamic hypothesis (Cardona, 2009), which suggests endogenous reinfection, into quantifiable and comparable models. Although we assumed a logistic growth of lesions, similar growth models (e.g., Gompertz model) with an initial exponential growth and an asymptotic maximum would provide similar results.

The ultimate aim of this model is to achieve a complete understanding of the mechanisms that prevent a TB infection from evolving toward active disease or accelerating this process. Therefore, an updated model should be capable of describing both a latent infection and active disease by simply changing some of the parameters or physical constraints involved. At this point, the bubble model correctly describes active disease in C3HeB/FeJ mice, an animal that reacts to Mtb with an exaggerated inflammatory response, and could easily be calibrated with data from similar experiments with mice. In fact, calibration of this model using experimental data from an animal model of latent infection should be the following step. Nevertheless, this will probably require inclusion of the immune response in the growth curve for individual lesions and the consideration of calcification dynamics.

Our results did not reveal a relevant function of mouse lung geometry. They are structurally simple, without septa inside, and they are sufficiently small to allow a rapid infection of the whole space. Nevertheless, in order to progress toward an understanding of the natural history of TB in the human body, the geometry of more complex lungs should be taken into account. In this sense, the role of spatial structures such as the bronchial tree in the endogenous reinfection process, or intralobular septae and encapsulation of the lesions (Gil et al., 2010; Cardona, 2015), should be tackled. Animal models such as minipigs or goats could provide valuable information about the role of lung geometry on the progression of TB (Parent, 2015). In a similar way to the hypothesis tests presented in this paper, the implementation of such a geometry in the mathematical model should allow an assessment of its relevance.

## Author contributions

CP designed and implemented the model, designed and carried out the simulations and *in silico* experiments, analyzed, and discussed the results and leaded the writing of the manuscript. CV analyzed experimental data, designed the model and the *in silico* experiments, analyzed and discussed the results, and critically revised the manuscript. JV designed and implemented the model, analyzed and discussed the obtained results, and critically revised the manuscript. EM analyzed experimental data, designed the model, and critically revised the manuscript. PC conceived and co-leaded this research project, analyzed experimental data, designed the model and the *in silico* experiments, analyzed and discussed the obtained results, and participated in the writing of the manuscript. DL conceived and leaded this research project, designed the model and the *in silico* experiments, analyzed and discussed the obtained results, and participated in the writing of the manuscript.

### Conflict of interest statement

The authors declare that the research was conducted in the absence of any commercial or financial relationships that could be construed as a potential conflict of interest.
